# Tehranolide Attenuates Lipid Accumulation in Steatotic HepG2 Cells via cAMP/AMPK/SIRT1-Mediated Autophagy Activation

**DOI:** 10.5812/ijpr-168037

**Published:** 2026-01-20

**Authors:** Shima Lorestani, Shahla Mohammad Ganji, Shokoofe Noori

**Affiliations:** 1 Department of Clinical Biochemistry, Faculty of Medicine, Shahid Beheshti University of Medical Sciences, Tehran, Iran; 2 Department of Molecular Medicine, National Institute of Genetic Engineering and Biotechnology, Tehran, Iran; 3 Department of Biochemistry, Faculty of Medicine, Shahid Beheshti University of Medical Sciences, Tehran, Iran

**Keywords:** Steatotic Cells, HepG2, Tehranolide, Autophagy, SIRT1, cAMP, AMPK, LC3

## Abstract

**Background:**

Metabolic dysfunction-associated steatotic liver disease (MASLD) is a prevalent metabolic condition marked by abnormal lipid buildup within hepatocytes, leading to inflammation and liver injury. Hepatic lipid accumulation can be driven by multiple factors, including high glucose, free fatty acids, and lipotoxic stress. Autophagy, which may be influenced by metabolic regulators such as cyclic adenosine monophosphate (cAMP), AMP-activated protein kinase (AMPK), and sirtuin 1 (SIRT1), is suggested to contribute to the maintenance of hepatic lipid homeostasis. Tehranolide, a sesquiterpene lactone derived from *Artemisia diffusa* and structurally related to artemisinin, is believed to have hepatoprotective effects similar to artemisinin. This work is the first to assess the impact of tehranolide on lipid accumulation with emphasis on autophagy/AMPK/SIRT1 signaling in steatotic human hepatoma-derived cells (HepG2).

**Objectives:**

This investigation was undertaken to evaluate the potential of tehranolide to reduce lipid accumulation in a high-glucose-induced steatotic hepatocyte model, potentially involving autophagy-related signaling pathways such as cAMP, AMPK, and SIRT1.

**Methods:**

A high-glucose–induced steatotic model was established in HepG2 cells. After determining the effective concentration of tehranolide by means of the MTT assay, lipid-loaded cells received treatment with tehranolide. The content of intracellular triglycerides (TGs) was determined via Oil Red O staining and commercial kits. The expression of lipid metabolism-related genes [fatty acid synthase (FASN), sterol regulatory element-binding protein 1c (SREBP-1c), and SIRT1] and autophagy markers [light chain 3 (LC3), beclin-1] was analyzed by quantitative real-time polymerase chain reaction (qRT-PCR), while protein levels of LC3-I, LC3-II, AMPK, and phosphorylated AMP-activated protein kinase (p-AMPK) were evaluated by Western blotting. Intracellular cAMP levels, lactate dehydrogenase (LDH) release, and inflammatory cytokines were also quantified using commercial kits.

**Results:**

Tehranolide significantly decreased intracellular TG levels, downregulated lipogenic genes (FASN, SREBP-1c), and upregulated the lipolytic gene SIRT1. It increased the expression of autophagy-related markers (beclin-1 and LC3-II). Furthermore, tehranolide increased intracellular cAMP and AMPK phosphorylation, while inhibition of SIRT1 or blockade of autophagy attenuated these effects. In addition, tehranolide reduced glucose-induced cytotoxicity and suppressed pro-inflammatory cytokine production in HepG2 cells.

**Conclusions:**

Tehranolide attenuates lipid accumulation and inflammatory responses in high-glucose-induced steatotic HepG2 cells, potentially involving autophagy-related processes, which may be linked to cAMP, AMPK, and SIRT1. These findings suggest that tehranolide may represent a potential modulator of hepatocellular lipid metabolism in glucose-induced steatosis, warranting further validation in more comprehensive in vitro and in vivo models.

## 1. Background

This study is the first to investigate the potential effects of tehranolide on glucose-induced lipid accumulation in HepG2 cells, an in vitro model potentially involving autophagy-related processes linked to cyclic adenosine monophosphate (cAMP), AMP-activated protein kinase (AMPK), and sirtuin 1 (SIRT1), used to study certain aspects of metabolic dysfunction-associated steatotic liver disease (MASLD), a condition affecting about 30% of the global population (1). The MASLD is a prevalent non-alcoholic liver disease distinguished by lipid overload in liver cells. Often linked to overweight/obesity, insulin dysfunction, and type 2 diabetes mellitus, it is usually asymptomatic but can progress from uncomplicated steatosis to steatohepatitis and liver fibrosis, cirrhosis, or liver cancer (2). Given its rising prevalence, particularly among overweight, diabetic, and sedentary individuals, and its potential to progress to liver failure, early diagnosis and treatment of MASLD are crucial. Currently, no definitive therapy exists; lifestyle interventions, including dietary modification control, weight loss, and exercise, remain the mainstay. Limited pharmacological options, including vitamin E and insulin-sensitizing agents like pioglitazone, are recommended, though their use is restricted to specific patient groups (3). Given the significance of MASLD, developing new complementary therapies to enhance or replace current treatments is crucial.

Autophagy, a key cellular mechanism for degrading and recycling damaged components, plays a vital role in maintaining liver homeostasis. Its dysfunction contributes to MASLD progression, as impaired autophagy hinders lipid droplet clearance, leading to excessive hepatic fat accumulation (4). Several factors regulate autophagy, with SIRT1 playing a key role. SIRT1, one of the sirtuin proteins, is a nicotinamide adenine dinucleotide (NAD^+^)-dependent enzyme with deacetylase activity that serves as an intracellular energy sensor and regulates various metabolic processes. It reduces hepatic lipid accumulation by controlling genes involved in lipid synthesis, including fatty acid synthase (FASN) and sterol regulatory element-binding protein 1c (SREBP-1c) (5). It also modulates autophagy-associated genes, such as microtubule-associated protein 1 light chain 3 (LC3) and beclin-1 (6). Therefore, SIRT1 is considered a potential therapeutic target to slow MASLD progression.

The SIRT1 and the energy sensor AMPK have a mutual regulatory relationship, affecting downstream signaling that controls various biological processes. Under low energy conditions, AMPK activation has been associated with increased NAD^+^ levels, which may enhance SIRT1 activity (7). One key outcome of this activation is the suppression of genes regulating lipid synthesis and the induction of autophagy-related genes in the liver. Intracellular adenylate cyclases are involved in signaling networks that are associated with AMPK and SIRT1 activation (8). The pathogenesis of MASLD is critically influenced by inflammatory signaling molecules such as interleukin-6 (IL-6), tumor necrosis factor-alpha (TNF-α), and interleukin-1 beta (IL-1β) (9). Additionally, SIRT1 deletion in liver cells inhibits fatty acid β-oxidation, promoting hepatic fat accumulation and subsequent inflammation (10).

**Figure 1.**
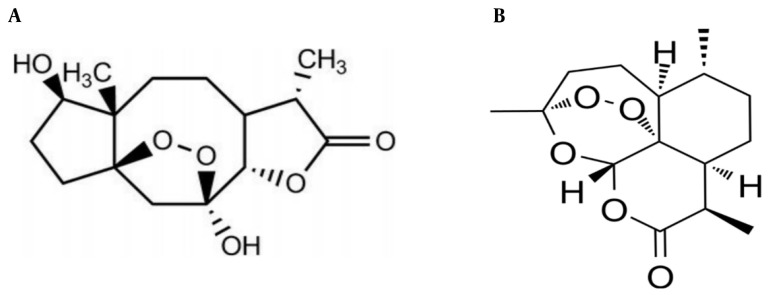
Molecular structure of tehranolide (A); molecular structure of artemisinin (B).

In recent decades, medicinal plants have gained attention as potential treatments for fatty liver disease. Tehranolide, a sesquiterpene lactone with an endoperoxide bond, is derived from the Iranian plant *Artemisia diffusa* and has a structure similar to artemisinin (Figure 1) (11).

Artemisinin, a sesquiterpene lactone with an endoperoxide bond derived from *A. annua*, has potent anti-malaria activity due to its endoperoxide bridge and is United States Food and Drug Administration (FDA)-approved as a first-line treatment worldwide (12). In 2015, Chinese researcher Youyou Tu was honored with the Nobel Prize in Physiology or Medicine for discovering the anti-malaria properties of artemisinin and for reducing mortality in patients with malaria (13). The results of numerous studies indicate that artemisinin contributes to liver health by regulating the expression of lipogenic and lipolytic genes, inhibiting inflammation and fibrosis, inducing apoptosis and autophagy, and reducing oxidative stress. Artemisinin affects various signaling cascades, among which autophagy-related processes potentially linked to AMPK and SIRT1 may play a role (14).

Studies have demonstrated that tehranolide, similar to artemisinin (15), significantly suppresses cancer cell proliferation, attenuates inflammation, and modulates immune responses (11, 16, 17). Due to the structural similarity between tehranolide and artemisinin, it is hypothesized that tehranolide exerts effects similar to those of artemisinin. Given the involvement of AMPK phosphorylation, SIRT1 activity, autophagy, cAMP signaling, and inflammatory responses in hepatic lipid accumulation, these pathways are explored in the context of glucose-induced steatosis in HepG2 cells. Although this cellular model does not fully replicate the complex pathophysiology of MASLD, it provides a controlled system to evaluate molecular mechanisms underlying glucose-driven hepatic lipid accumulation and potential therapeutic interventions.

## 2. Objectives

This study is the first to investigate the effects of tehranolide on lipid accumulation in a high-glucose–induced cellular model of hepatic steatosis. The study focused on autophagy-related pathways potentially associated with cAMP, AMPK, and SIRT1, which are involved in hepatic metabolic homeostasis. The aim was to evaluate the association between tehranolide treatment and modulation of autophagy-related markers, as well as to explore potential molecular targets relevant to glucose-driven hepatocellular steatosis. The findings showed that tehranolide significantly reduced intracellular lipid accumulation, accompanied by increased cAMP levels, AMPK phosphorylation, and SIRT1 expression, along with upregulation of autophagy-related markers in steatotic HepG2 cells.

## 3. Methods

### 3.1. Chemicals

Tehranolide, extracted and purified from *A. diffusa*, whose structural integrity was confirmed by 13C nuclear magnetic resonance (NMR) spectroscopy, was supplied by the Faculty of Chemistry, Shahid Beheshti University (Tehran, Iran). Other reagents included Dulbecco's Modified Eagle Medium (DMEM; DB9696), dimethyl sulfoxide (DMSO; DB9693), Oil Red O solution (Bioidea), 1X radioimmunoprecipitation assay (RIPA) buffer (DB9719), protease inhibitor cocktail (P8340), sirtinol (Sigma-Aldrich), bafilomycin A1 (BafA1; Merck, Germany), forskolin (F6886), KH7, compound C (Sigma-Aldrich), among others. The main antibodies recognizing microtubule-associated protein LC3 (sc-271625), β-actin, AMPK (sc-74461), and phosphorylated AMP-activated protein kinase (p-AMPK; sc-33524) were obtained from Santa Cruz Biotechnology.

### 3.2. Cell Culture

Human HepG2 cells were obtained from the Pasteur Institute of Iran (Tehran). The cells were grown in DMEM (DNAbiotech, Gibco) enriched with 10% fetal bovine serum (FBS) (DNAbiotech, Iran) and 100 U/ml penicillin–streptomycin. The cultures were grown at 37 °C with 5% CO_2_ and controlled humidity (18).

### 3.3. Establishment of a High-Glucose-Induced Steatotic HepG2 Cell Model

HepG2 cells were introduced into 12-well plates at a seeding density of 1.2 × 10^6^ cells per well, and left to settle and attach overnight under standard incubator conditions. On the subsequent day, the growth medium was switched to serum-free DMEM, and the cells were pre-incubated for 12 hours. Filtered glucose solutions at final concentrations of 20 and 35 mM were applied to the wells. Following 24 hours of treatment, the supernatant was discarded, and the cells underwent Oil Red O staining to visualize lipid accumulation (19).

### 3.4. Lipid Content Analysis

To validate the induction of steatosis, lipid levels in HepG2 cells were assessed using Oil Red O staining. After glucose treatment (20 and 35 mM), fixation of the cells was carried out using 10% formalin for 5 minutes and left at room temperature for 1 hour. A working staining solution was obtained by blending Oil Red O stock and distilled water in a 6:4 proportion. Plates were washed three times with 60% isopropanol, air-dried, incubated with the filtered solution for 10 minutes, subsequently washed with distilled water and allowed to dry naturally, then observed under an inverted microscope (20).

### 3.5. Measurement of Triglyceride Content

After treating HepG2 cells with 20 and 35 mM glucose solutions for 24 hours, the intracellular triglyceride (TG) levels were measured using a TG assay kit. In addition, HepG2 cells were treated with 35 mM filtered glucose (effective concentration of glucose) for 24 hours, followed by tehranolide (5 - 25 μM) in the presence of BafA1, for 24 hours. Then, the TG levels were measured using a commercial assay kit. To measure intracellular TGs, after washing with phosphate-buffered saline (PBS), the cells were lysed on ice for 5 minutes in 1X RIPA buffer [50 mM Tris-HCl, pH 7.4, 1% Triton X-100, 0.2% sodium deoxycholate, 0.2% sodium dodecyl sulfate (SDS), 1 mM Na-EDTA, 1 mM phenylmethylsulfonyl fluoride (PMSF)] with protease inhibitors (1:100), and centrifuged for 10 minutes. The supernatants were collected, kept at 4 °C, and TGs were quantified enzymatically using a commercial kit (Pars-Azmoon, Iran). Normalization was performed using total protein content quantified using the bicinchoninic acid (BCA) method (21).

### 3.6. Cytotoxicity Assessment

Cellular toxicity of tehranolide was assessed using an MTT colorimetric assay. For this purpose, HepG2 cells were seeded at a density of 1 × 10^4^ cells per well in 96-well plates and incubated for 24 hours at 37 °C in a humidified atmosphere containing 5% CO_2_. After 12 hours of serum starvation, the cells were exposed to a final concentration of 35 mM glucose for 24 hours. After this period, cells were then treated with tehranolide (5 - 120 µM) in quintuplicate for 24 hours. Cells were treated with 20 µL of MTT (5 mg/mL) and incubated for 4 hours; subsequently, formazan crystals were dissolved in 50 µL DMSO. Absorbance at 570 nm was measured after 15 minutes, and cell viability was expressed relative to untreated steatotic controls. Glucose cytotoxicity (20 - 75 mM, 24 hours) was also evaluated using the MTT assay (22).

### 3.7. Real-Time Polymerase Chain Reaction

After treatment of steatotic HepG2 cells with tehranolide, cellular RNA was isolated using an RNA purification kit (Parstous, Iran). Subsequently, complementary DNA (cDNA) was generated with the cDNA synthesis kit (Parstous, Iran). The expression levels of FASN, SREBP-1c, SIRT1, microtubule-associated protein LC3, and beclin-1 genes were evaluated through real-time polymerase chain reaction (PCR) with SYBR Green Master Mix (Parstous, Iran) and primers sourced from Metabion (Germany). Gene expression data were normalized against β-actin and quantified utilizing the 2^-ΔΔCt^ method. The sequences of the primers can be found in Table 1 (23).

**Table 1.** Real-time PCR Primer Sequences

Gene Names	Forward Primer (5′-3′)	Reverse Primer (5′-3′)
**FASN**	GTGAGGCTGAGGCTGAGAC	GGCACGCAGCTTGTAGTAGA
**SIRT1**	TGCTGGCCTAATAGAGTGGCA	CTCAGCGCCATGGAAAATGT
**SREBP-1c**	CCATGGATTGCACTTTCGAA	GGCCAGGGAAGTCACTGTCTT
**LC3**	AAGGCGCTTACAGCTCAATG	CTGGGAGGCATAGACCATGT
**Beclin-1**	AGCTGCCGTTATACTGTTCTG	ACTGCCTCCTGTGTCTTCAATCTT
**β-actin**	AAGGCCAACCGCGAGAAGAT	GCCAGAGGCGTACAGGGATA

### 3.8. Assessment of Inflammatory Cytokines

Following treatment of steatotic HepG2 cells with tehranolide, concentrations of cytokines IL-1β, TNF-α, and IL-6 in the cell culture supernatant were quantified through the use of enzyme-linked immunosorbent assay (ELISA) kits (Karmia Pars Gene, Iran). A calibration curve was employed to calculate cytokine concentrations (24).

### 3.9. Western Blot Assay

Glucose-stimulated steatotic HepG2 cells treated with tehranolide were rinsed with chilled PBS, disrupted in RIPA lysis buffer with protease inhibitors on ice for 5 minutes, and centrifuged. Total protein levels were quantified using the BCA assay, and equal amounts of protein (40 μg per sample) were separated on 10% sodium dodecyl sulfate-polyacrylamide gel electrophoresis (SDS-PAGE) and electrotransferred onto polyvinylidene difluoride (PVDF) membranes. Membranes were blocked in 5% non-fat milk for 1 hour at room temperature, overnight incubation at 4 °C was performed with primary antibodies recognizing LC3-I/II, AMPK, p-AMPK, and β-actin, followed by 1 hour incubation with secondary antibodies in the dark. Signal detection was performed using a chemiluminescence kit, and intensities were quantified with ImageJ (25).

### 3.10. Cyclic Adenosine Monophosphate Level Assessment

Treatment with tehranolide was performed in steatotic HepG2 cells both with and without forskolin (10 µM), an adenylate cyclase stimulator (26), and KH7 (10 µM), an adenylate cyclase inhibitor (27). Subsequently, total intracellular cAMP levels in lysates from steatotic HepG2 cells were determined using an enzymatic immunoassay kit (Cell Biolabs, USA). In this assay, only a limited number of antibody binding sites are available for cAMP, and competition occurs between unlabeled and peroxidase-conjugated cAMP molecules for these sites. The final concentration was normalized to the total intracellular protein (21).

### 3.11. Lactate Dehydrogenase Release Analysis

Cell death was induced in HepG2 cells by exposure to 75 mM glucose. To evaluate the effect of tehranolide on glucose-mediated cell toxicity, cells were treated with 25 µM tehranolide with and without rapamycin (Rapa; 100 nM), sirtinol (100 µM), and BafA1 (100 nM). Sirtinol functions as a specific inhibitor of the SIRT1 enzyme (28). Baf inhibits the fusion of lysosomes with autophagosomes, thereby preventing the maturation of autophagic vesicles (29). Conversely, Rapa stimulates the initiation of autophagy by inhibiting mammalian target of rapamycin (mTOR) kinase activity (30). Initially, cells were treated with 75 mM glucose for 24 hours. Subsequently, the designated treatments were applied, and the supernatant was harvested to assess lactate dehydrogenase (LDH) release. The LDH activity, as a biomarker of cell damage, was measured using an LDH assay kit (Kiazist, Iran). Absorbance was recorded at 546 nm using an ELISA reader (31).

### 3.12. Statistical Analyses

Statistical analyses were conducted using GraphPad Prism v8.0.2. One-way analysis of variance (ANOVA) followed by Tukey’s multiple comparisons test was used to compare differences among groups. Every assay was conducted in triplicate and repeated independently no fewer than three times. Data are shown as mean ± standard deviation (SD), with statistical significance considered at P < 0.05.

## 4. Results

### 4.1. Establishment of a High-Glucose-Induced Steatotic HepG2 Cell Model

**Figure 2.**
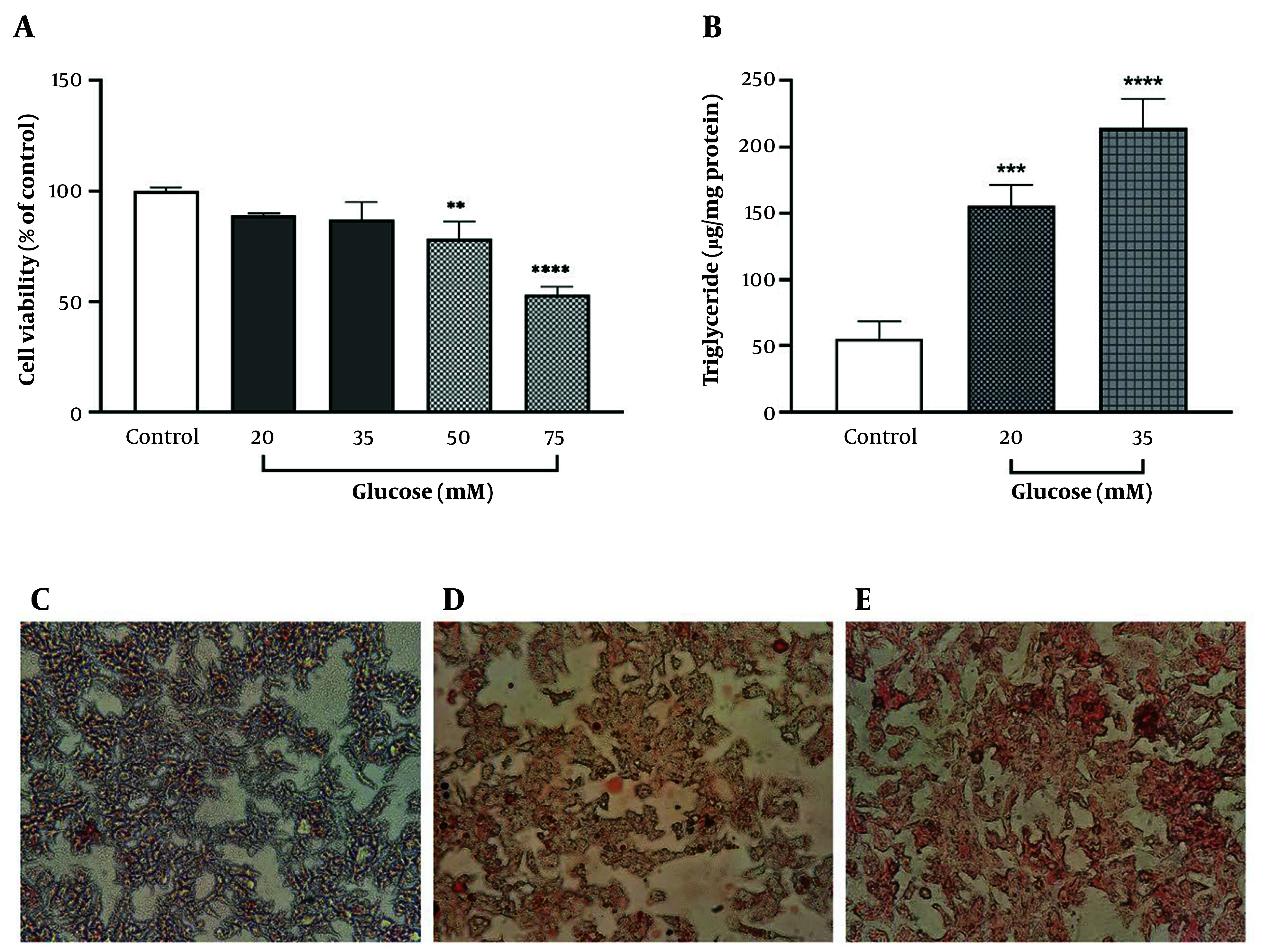
Cell viability assay of HepG2 cells treated with glucose solutions showed no cytotoxicity at 20 and 35 mM concentrations, whereas significant cytotoxic effects were observed at 50 and 75 mM (A). HepG2 cells were incubated with 20 and 35 mM glucose. Quantitative measurement using a triglyceride (TG) assay kit confirmed a significant increase in lipid content at both concentrations (B). Following Oil Red O staining, HepG2 cells in three groups were observed under an inverted microscope: Untreated cells (C), treated cells with 20 mM glucose (D), treated cells with 35 mM glucose (E). Data are presented as mean ± standard deviation (SD) of three independent biological experiments, each performed in triplicate (technical replicates). One-way analysis of variance (ANOVA) was conducted followed by Tukey’s post-hoc test for multiple comparisons (** P < 0.01; *** P < 0.001; **** P < 0.0001).

HepG2 cells were exposed to 20 - 75 mM glucose for 24 hours to assess cytotoxicity via MTT assay. No cytotoxicity was observed at 20 and 35 mM, while 50 and 75 mM caused significant cell death (Figure 2A). Lipid accumulation was induced using 20 and 35 mM glucose following 12 hours of serum starvation. Intracellular TGs were quantified with a commercial assay kit (Figure 2B) and visualized by Oil Red O staining (Figure 2C - E). As shown in Figure 2B, glucose treatment significantly increased lipid content compared to controls, with 35 mM inducing higher levels than 20 mM; thus, 35 mM was selected for steatosis induction in subsequent experiments.

### 4.2. Tehranolide Decreases Lipid Deposition in Glucose-Induced Steatotic HepG2 Cells

**Figure 3.**
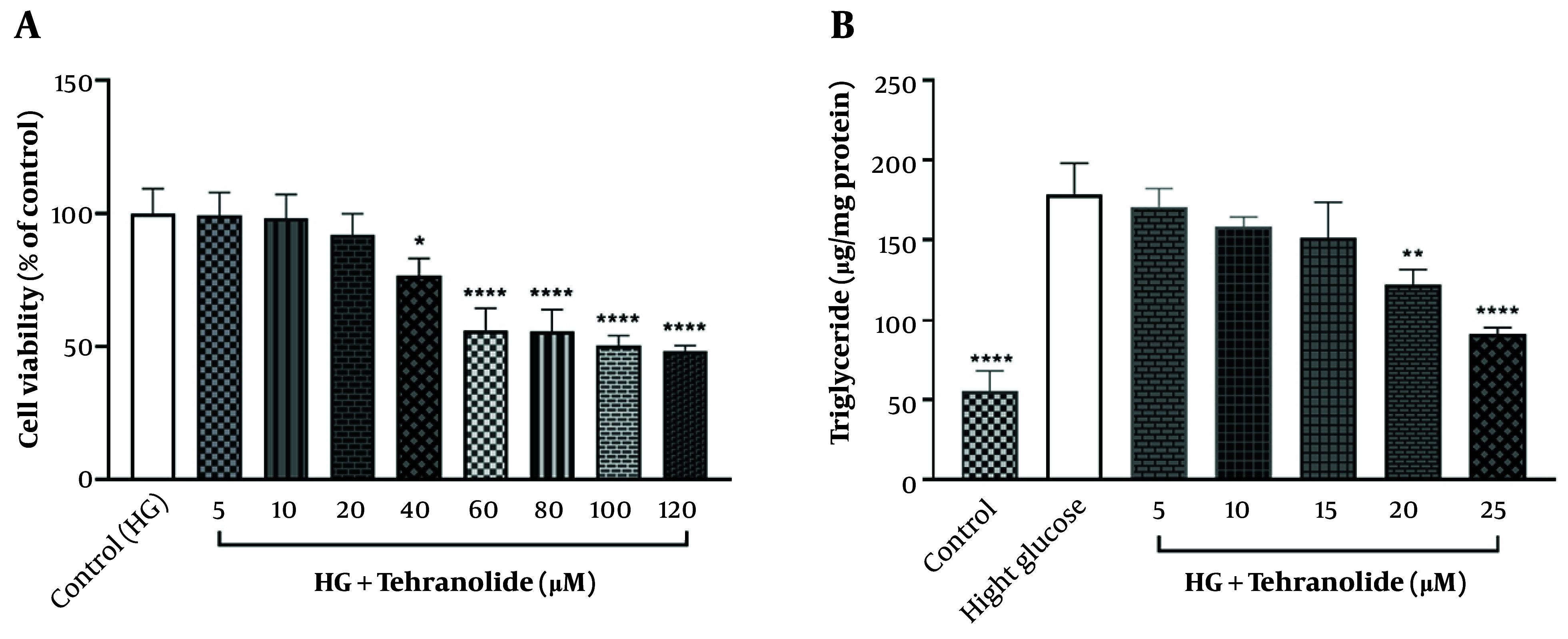
The MTT assay was performed after 24 hours of treatment of steatotic HepG2 cells with varying concentrations of tehranolide. No significant cytotoxicity was observed at concentrations below 40 µM, whereas cell viability decreased significantly at higher concentrations (A). Intracellular triglyceride (TG) levels in steatotic HepG2 cells were measured in response to various concentrations of tehranolide using a TG assay kit. No statistically significant changes in TG levels were observed at tehranolide concentrations of 5 - 15 µM compared to untreated steatotic HepG2 cells (high glucose group), however, significant reductions were observed at 20 and 25 µM (B). Data are presented as mean ± standard deviation (SD) of three independent biological experiments, each performed in five technical replicates. Statistical analysis was conducted using one-way analysis of variance (ANOVA) followed by Tukey’s post-hoc test for multiple comparisons [* P < 0.05; ** P < 0.05; **** P < 0.0001; abbreviations: HG, high glucose (35 mM)].

Prior to further experiments, safe concentrations of tehranolide in glucose-induced steatotic HepG2 cells were evaluated through the MTT method. Steatosis in HepG2 cells was induced with 35 mM glucose (high glucose) and subsequently the cells were treated with tehranolide (5 - 120 µM) for 24 hours. The MTT assay, compared to the control group (untreated glucose-induced steatotic HepG2 cells), revealed a dose-dependent decrease in viability, with significant cytotoxicity above 40 µM, while concentrations below 40 µM showed minimal effect (Figure 3A). IC_50_, IC_10_, and IC_15_ were determined, and due to minimal cytotoxicity and effective TG reduction at IC_10_ (~20 µM) and IC_15_ (~25 µM, Figure 3B), these concentrations were chosen for further experiments. To determine the role of tehranolide on lipid accumulation, HepG2 cells with glucose-induced steatosis were treated with tehranolide at doses in the range of 5 to 25 µM for 24 hours. Intracellular TG levels were measured in cell lysates and normalized to total intracellular protein. The results showed that tehranolide at concentrations of 20 and 25 µM significantly reduced TG levels compared to untreated glucose-induced steatotic HepG2 cells (high glucose group), whereas no significant effect was observed at 5 to 15 µM (Figure 3B). 

### 4.3. Tehranolide Alters Lipid Metabolism Through the Activation of Lipolytic Pathways and Suppression of Lipogenic Pathways

**Figure 4.**
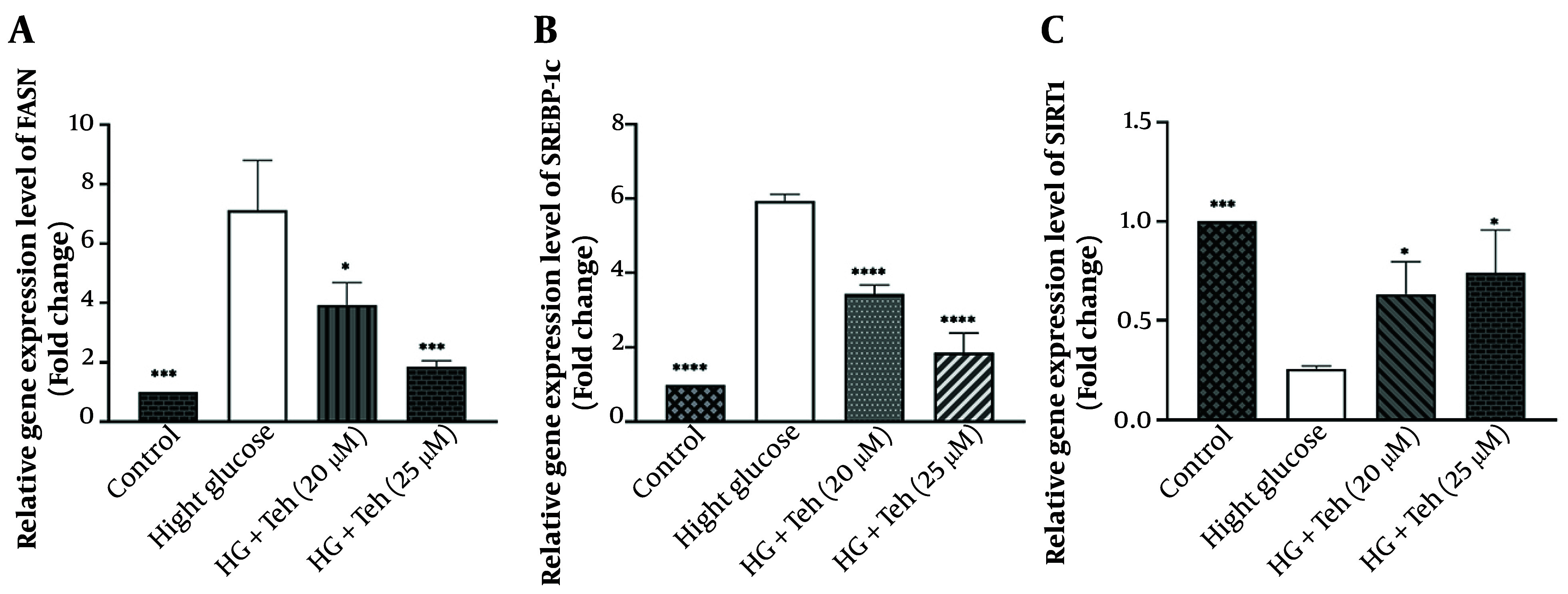
Evaluation of fatty acid synthase (FASN) (A), sterol regulatory element-binding protein 1c (SREBP-1c) (B), and sirtuin 1 (SIRT1) (C) gene expression levels in glucose-induced steatotic HepG2 cells following treatment with 20 and 25 µM tehranolide was performed using real-time polymerase chain reaction (PCR). Tehranolide treatment led to a significant downregulation of the lipogenic genes FASN and SREBP-1c, accompanied by an upregulation of the lipolytic gene SIRT1. Data are presented as mean ± standard deviation (SD) of three independent biological experiments, each conducted in duplicate as technical replicates. Statistical analysis was carried out using one-way analysis of variance (ANOVA) followed by Tukey’s post-hoc test for multiple comparisons [* P < 0.05; *** P < 0.001; **** P < 0.0001; abbreviations: HG, high glucose (35 mM); Teh, tehranolide].

The effects of tehranolide on SIRT1, FASN, and SREBP-1c expression were assessed in glucose-induced steatotic HepG2 cells. Cells were treated with 20 and 25 µM tehranolide for 24 hours, followed by RNA extraction, cDNA synthesis, and real-time PCR analysis using the 2^-ΔΔCt^ method with melting curve confirmation. Compared to control (normal cells), untreated steatotic cells showed significant upregulation of FASN and SREBP-1c and downregulation of SIRT1. Tehranolide treatment reduced FASN and SREBP-1c expression and increased SIRT1 at both concentrations of tehranolide (Figure 4A - C).

### 4.4. Tehranolide Activates Sirtuin 1-Dependent Autophagy Thereby Attenuates Hepatic Steatosis in Glucose-Induced HepG2 Cells

**Figure 5.**
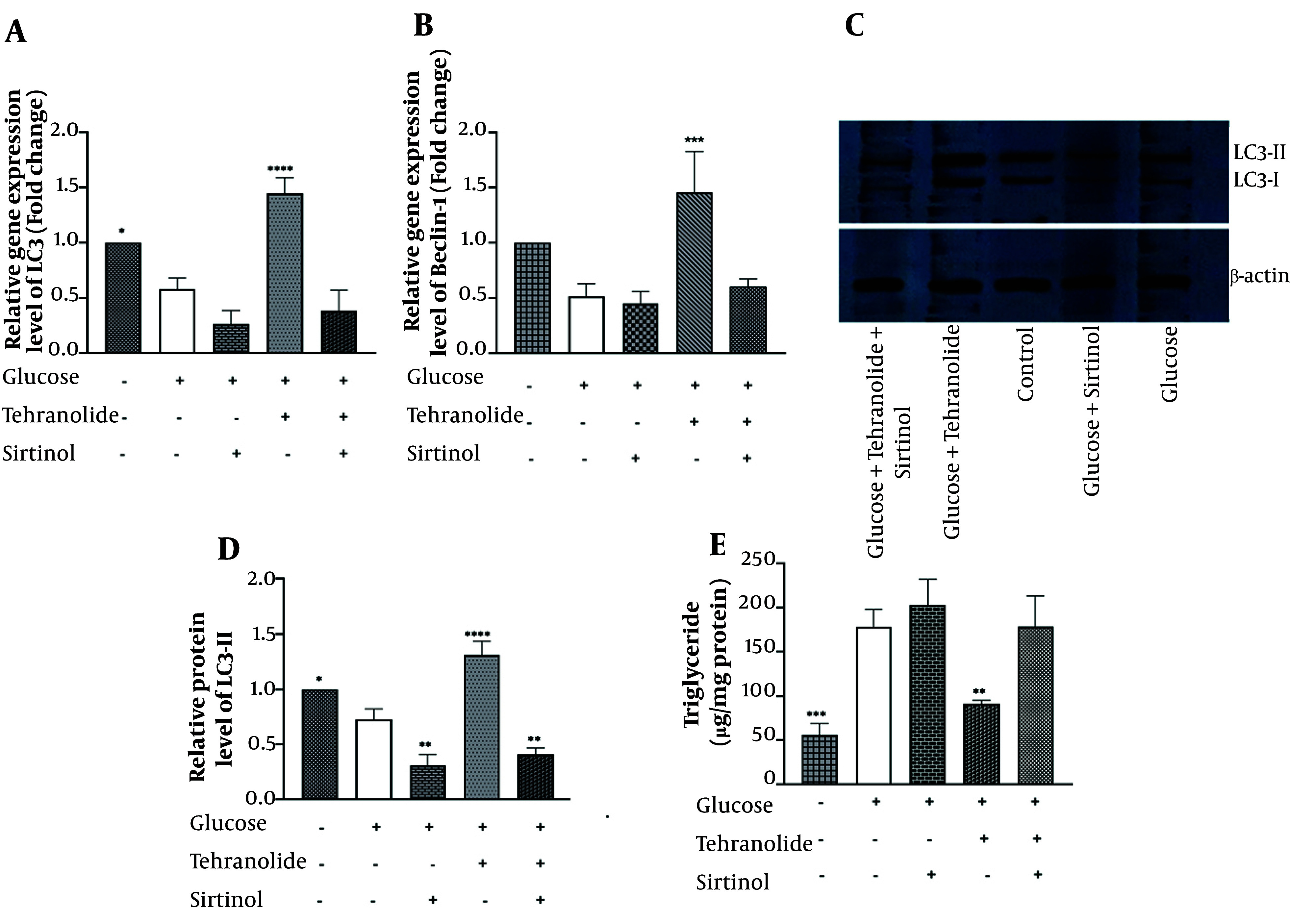
Tehranolide induces sirtuin 1 (SIRT1)-dependent autophagy in a HepG2 steatosis model. Real-time polymerase chain reaction (PCR) was used to assess autophagy-related gene expression. Tehranolide (25 µM) significantly increased LC3 and beclin-1 mRNA levels, while co-treatment with the SIRT1 inhibitor sirtinol attenuated these effects (A and B). Western blot confirmed autophagy induction by elevated LC3-II levels (C and D). Tehranolide also reduced intracellular triglycerides (TGs), and this effect was reversed by the autophagy inhibitor bafilomycin A1 (BafA1), indicating autophagy-dependent TG reduction (E). All data are presented as mean ± standard deviation of three independent biological experiments, each performed in triplicate as technical replicates. Statistical comparisons were made using one-way analysis of variance (ANOVA) followed by Tukey’s post-hoc test for multiple comparisons, with significance defined as *P < 0.05, **P < 0.01, ***P < 0.001, and ****P < 0.0001.

The effect of tehranolide (25 µM) on autophagy was assessed in steatotic HepG2 cells by measuring beclin-1 and microtubule-associated protein LC3 mRNA levels via real-time PCR. Lipid accumulation caused a slight, non-significant decrease in beclin-1 and a significant reduction in LC3 compared to normal cells. Tehranolide treatment significantly upregulated both genes. To evaluate SIRT1 involvement, cells were treated with the SIRT1 inhibitor sirtinol (100 µM), which markedly blocked tehranolide-induced beclin-1 and LC3 upregulation, indicating SIRT1 dependency (Figure 5A and B). Additionally, Western blot analysis was performed to measure the expression of LC3 isoform proteins (LC3-I and LC3-II). During autophagy, LC3 undergoes lipidation, converting cytosolic LC3-I to membrane-associated LC3-II, which is recruited to autophagosomal membranes (32). Western blotting confirmed increased LC3-I and LC3-II protein levels after tehranolide treatment, consistent with enhanced autophagy, while sirtinol (100 µM) attenuated this effect (Figure 5C and D). The role of autophagy in tehranolide-mediated lipid reduction was further confirmed using BafA1 (100 nM) to inhibit autophagic flux. Tehranolide significantly reduced intracellular TGs in steatotic cells compared to the positive control group (the group treated with glucose alone; P < 0.01), whereas this effect was abolished in cells treated with both tehranolide and Baf, showing no statistically significant difference from the positive control group. Steatotic cells treated with Baf alone exhibited an increase in intracellular TGs compared to the positive control, although this increase was not statistically significant. These results suggest that the anti-steatotic effect of tehranolide may be dependent on autophagy induction.

### 4.5. Tehranolide Activates AMP-Activated Protein Kinase Phosphorylation via a Cyclic Adenosine Monophosphate-Dependent Pathway in Glucose-Induced Steatotic HepG2 Cells

**Figure 6.**
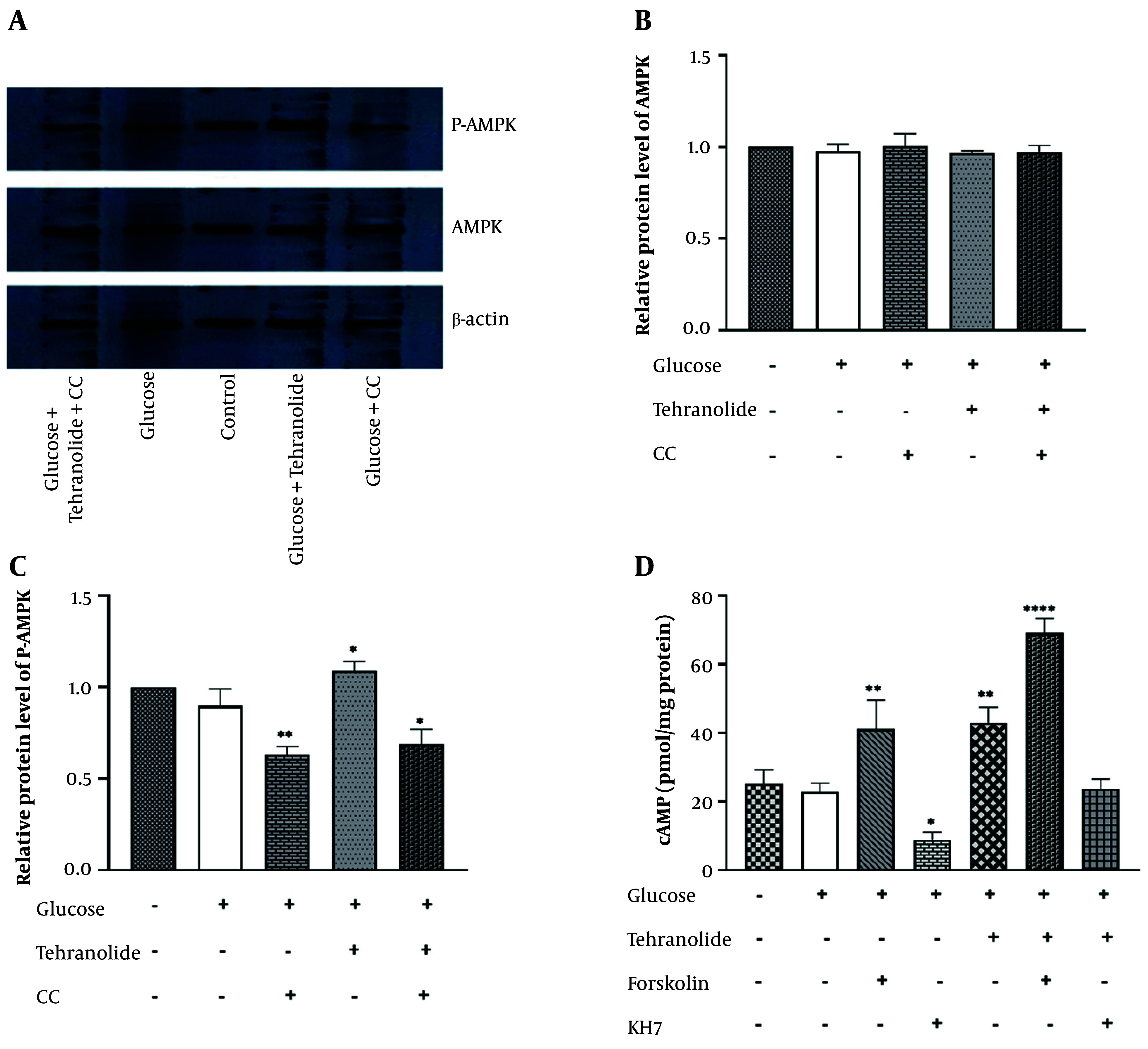
Cyclic adenosine monophosphate (cAMP) levels and AMP-activated protein kinase (AMPK) phosphorylation were increased by tehranolide in glucose-induced HepG2 cells. Western blot showed tehranolide (25 µM) had no effect on total AMPK (A and B) but increased p-AMPK, suppressed by compound C (A and C). For cAMP, glucose-stimulated cells treated with tehranolide (25 µM), with or without KH7 (10 µM) and forskolin (10 µM), showed elevated cAMP inhibited by KH7 (D). All data are presented as mean ± standard deviation of three independent biological experiments, each performed in triplicate as technical replicates. Statistical comparisons were made using one-way analysis of variance (ANOVA) followed by Tukey’s post-hoc test for multiple comparisons, with significance defined as * P < 0.05, ** P < 0.01, and **** P < 0.0001.

Western blot was used to assess the effect of tehranolide (25 µM) on AMPK levels and AMPK phosphorylation in glucose-induced steatotic HepG2 cells. No significant changes were observed in AMPK levels across the experimental groups. Glucose treatment did not affect p-AMPK levels, whereas tehranolide markedly increased p-AMPK expression. Compound C (CC), an AMPK inhibitor, was employed. CC binds to the adenosine triphosphate (ATP)-binding site of AMPK, preventing its phosphorylation (33). The results showed that CC (10 μM) suppressed the tehranolide-induced increase in AMPK phosphorylation (Figure 6A - C). Intracellular cAMP levels were measured after tehranolide treatment. As shown in Figure 6D, tehranolide significantly increased cAMP compared to untreated steatotic cells. As controls, KH7 (adenylyl cyclase inhibitor; 10 μM) and forskolin (adenylyl cyclase activator; 10 μM) were used. KH7 reduced, and forskolin further enhanced, the tehranolide-induced cAMP increase (Figure 6D). 

### 4.6. Induction of Autophagy by Tehranolide Confers Protection against High Glucose-Mediated Cell Death in HepG2 Cells

**Figure 7.**
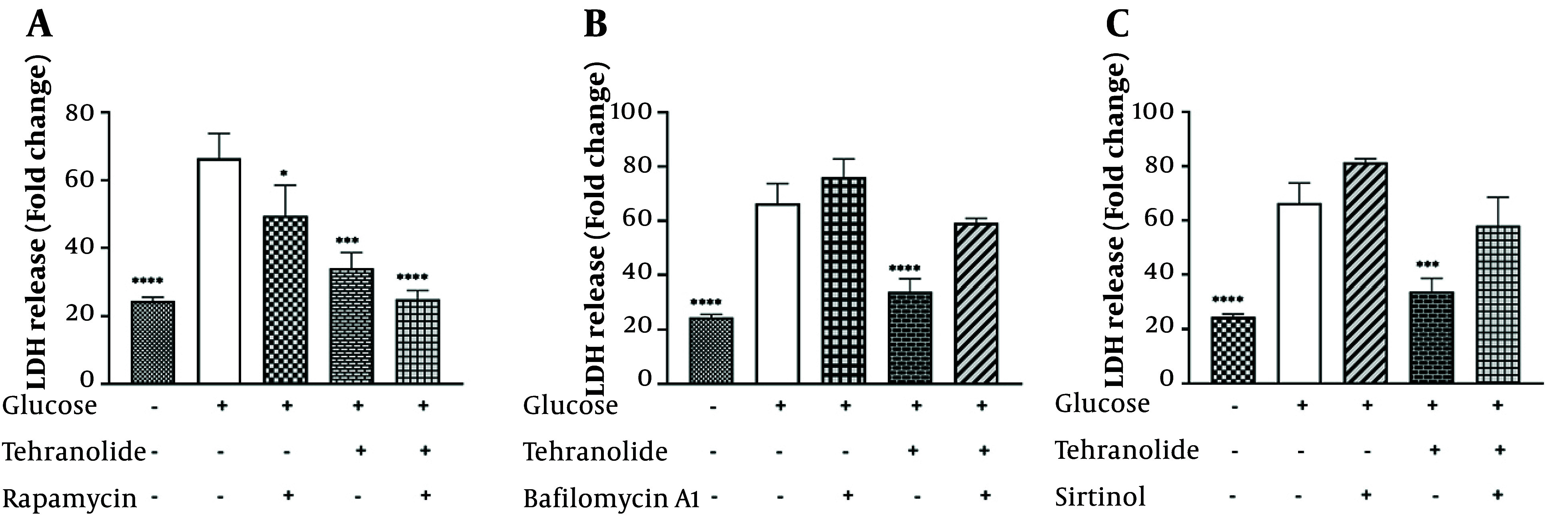
Tehranolide protects HepG2 cells from glucose-induced cytotoxicity via autophagy. Cells were exposed to high glucose (75 mM) with or without tehranolide (25 μM), rapamycin (100 nM), bafilomycin A1 (BafA1; 100 nM), or sirtinol (100 μM). The release of lactate dehydrogenase (LDH) was determined using an assay kit. Glucose markedly increased LDH, while tehranolide reduced it, showing protection. Rapamycin enhanced tehranolide’s effect (A), BafA1 abolished it (B), and sirtinol attenuated it, indicating SIRT1-dependent autophagy-mediated cytoprotection (C). All results are reported as mean ± standard deviation (SD) of three independent biological experiments, each performed in triplicate as technical replicates. Statistical comparisons were made using one-way analysis of variance (ANOVA) followed by Tukey’s post-hoc test for multiple comparisons, with significance levels defined as * P < 0.05, *** P < 0.001, and **** P < 0.0001.

The protective potential of tehranolide against cytotoxicity caused by high glucose exposure in HepG2 cells was evaluated using the LDH release assay. As shown in Figure 7, exposure to 75 mM glucose caused a strong increase in LDH release, indicating severe cytotoxicity (P < 0.0001). Tehranolide (25 μM) markedly reduced LDH release compared to the positive control group (the group treated with glucose alone), showing its protective effect and maintenance of cell integrity (P < 0.001). To assess autophagy involvement, modulators (rapamycin, BafA1, and sirtinol) were used. Cells under 75 mM glucose were treated with tehranolide ± rapamycin (100 nM), BafA1 (100 nM), or sirtinol (100 μM), and LDH was measured after 16 hours.

Rapamycin alone reduced LDH release in steatotic cells compared to the positive control group (P < 0.05), which was further increased by tehranolide, such that a statistically significant difference was observed between the group treated with both tehranolide and rapamycin and the positive control group (P < 0.0001), indicating a synergistic effect (Figure 7A). In contrast, BafA1 alone increased LDH compared to the positive control group, although this increase was not statistically significant; however, in combination with tehranolide, it abolished the protective effect of tehranolide, such that no statistically significant difference was observed between the group treated with both tehranolide and BafA1 and the positive control group (Figure 7B). Inhibition of SIRT1 by sirtinol reduced the tehranolide-mediated suppression of LDH, suggesting that its autophagy-dependent protective effect may be partially SIRT1-dependent. Sirtinol alone increased LDH compared to the positive control group, although this increase was not statistically significant; however, when combined with tehranolide, it blocked the protective effect of tehranolide, resulting in no statistically significant difference between the group treated with both tehranolide and sirtinol and the positive control group (Figure 7C). 

### 4.7. Tehranolide Mitigates Glucose-Induced Inflammatory Response in Steatotic HepG2 Cells via Suppression of Pro-inflammatory Cytokines

**Figure 8.**
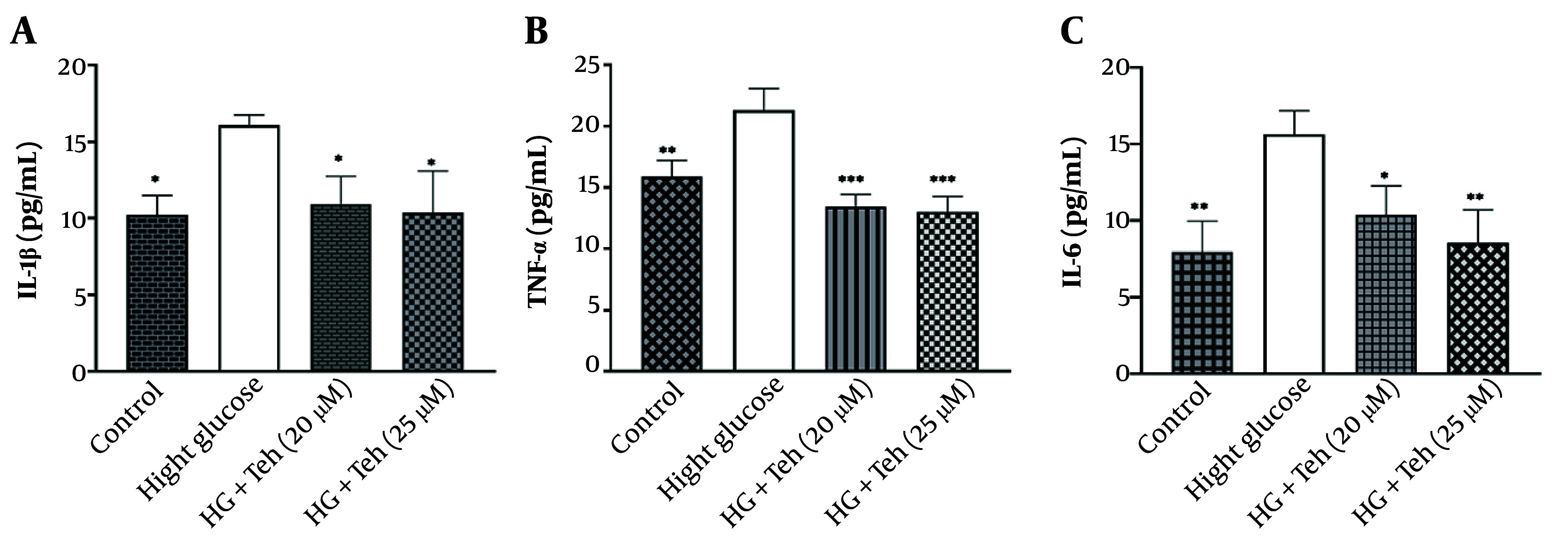
Using enzyme-linked immunosorbent assay (ELISA) assays, the effects of tehranolide on inflammatory cytokines were evaluated in a hepatic steatosis model. Cells were exposed to 35 mM glucose solution for 24 hours, followed by treatment with tehranolide at concentrations of 20 and 25 μM for 24 hours. The levels of interleukin-1 beta (IL-1β) (A), tumor necrosis factor-alpha (TNF-α) (B), and interleukin-6 (IL-6) (C) in the cell culture supernatant were measured. The results demonstrated that glucose significantly increased pro-inflammatory cytokines, while tehranolide treatment notably reduced the levels of these cytokines. The data represent the mean of three independent biological experiments; each performed in triplicate as technical replicates. Statistical analysis was performed using one-way analysis of variance (ANOVA), followed by Tukey’s post-hoc test for multiple comparisons, and the data are mean ± standard deviation [SD; * P < 0.05; ** P < 0.01; *** P < 0.001; abbreviations: HG, high glucose (35 mM); The, tehranolide].

The ELISA assays were conducted to evaluate the effects of tehranolide (20 and 25 μM) on glucose-induced inflammatory responses. The findings indicated that treatment of HepG2 cells with glucose significantly elevated the levels of pro-inflammatory cytokines. However, co-treatment with tehranolide at both tested concentrations markedly attenuated the expression of these cytokines (Figure 8). 

## 5. Discussion

The MASLD, marked by an abnormal accumulation of TG, is considered a widespread hepatic-related disorder with an increasing global prevalence (34). The excessive deposition of fat induces oxidative stress and triggers inflammatory responses, leading to hepatic tissue damage (35). The scarcity of efficient pharmacological agents for this disease (36) emphasizes the urgent requirement to discover innovative therapeutic methods. Evidence from multiple studies indicates that autophagy contributes significantly to lipid regulation in hepatic cells by mediating the degradation of lipid droplets. Impairment of this process can lead to excessive lipid deposition in hepatic tissue (37). Therefore, autophagy has emerged as an important cellular process implicated in lipid handling and metabolic homeostasis in hepatic cells, and is increasingly explored as a potential target in experimental models of hepatic steatosis. Components of the cAMP, AMPK, and SIRT1 pathways play a crucial role in hepatocellular lipid metabolism, potentially in part by modulating autophagy-related processes and coordinating the balance between the expression of lipogenic and lipolytic genes (38).

Building on this framework, the present study provides initial insights into the effects of tehranolide on lipid accumulation in a glucose-induced steatotic HepG2 cell model, with a focus on autophagy-related processes and pathways potentially involving cAMP, AMPK, and SIRT1. Tehranolide is a sesquiterpene lactone originating from *A. diffusa* and is structurally very similar to artemisinin. In the molecular structure of both, there is an endoperoxide bond responsible for their biological activities (11). According to previous studies, artemisinin exerts significant protective effects on liver tissue through various molecular mechanisms, encompassing the regulation of genes associated with lipid metabolic processes (39). Artemisinin exerts its effects by regulating various proteins and enzymes, including SIRT1, AMPK, SREBP-1c, FASN, and proteins associated with the autophagic pathway, including LC3-I, LC3-II, and beclin-1 (40-42). Due to its structural similarity to artemisinin, tehranolide is expected to have similar molecular effects, and this study, for the first time, explored the potential association between tehranolide treatment and modulation of lipid accumulation, possibly involving AMPK, SIRT1, and autophagy-related pathways.

To investigate these effects, a hepatic steatosis model was created by treating HepG2 cells with 35 mM glucose, a non-toxic concentration known to reliably elevate cellular TGs and induce steatosis. This range of glucose concentrations for inducing steatosis in HepG2 cells has been confirmed by previous studies (43). Two tehranolide concentrations with minimal toxicity and maximal TG reduction were selected for further assays, while a single concentration (25 µM) was used in experiments involving inhibitors or activators, including Western blot, cAMP, and LDH assays. In this high-glucose–induced steatotic HepG2 cell model, tehranolide significantly reduced TGs. The mRNA and protein expression levels of lipogenic genes, including FASN and SREBP-1c, the lipolytic gene SIRT1, and autophagy-related genes (microtubule-associated protein LC3 and beclin-1) were evaluated using real-time PCR and Western blot analyses.

High glucose increased FASN and SREBP-1c expression, which can be explained by the activation of mechanistic target of rapamycin complex 1 (mTORC1), as high glucose lowers the AMP/ATP ratio, suppresses AMPK, activates mTORC1, and upregulates lipogenic genes, promoting lipid accumulation (44). In contrast, tehranolide treatment of steatotic cells significantly reduced the expression of these genes. Similarly, previous studies have shown that artemisinin, structurally similar to tehranolide, also suppresses FASN and SREBP-1c mRNA levels (45, 46).

While high glucose decreased the expression of the lipolytic gene SIRT1, tehranolide increased its expression. Under high-glucose conditions, the NAD^+^/NADH ratio decreases, the AMPK pathway is inhibited, and the mTORC1 pathway is activated; collectively, these metabolic alterations can lead to a reduction in SIRT1 expression and activity (47). Concomitantly, real-time PCR showed that tehranolide significantly upregulated beclin-1 and LC3 expression in glucose-induced HepG2 cells, which may indicate increased involvement of autophagy-related processes. Beclin-1 serves as a key regulator at the initiation of autophagy (48), while LC3 is also a critical autophagy-associated protein (49).

Western blot and real-time PCR analyses demonstrated that 35 mM glucose reduced LC3 (LC3-I and LC3-II) protein levels. This may reflect that, under nutrient-rich conditions like high glucose, cells require less autophagy due to abundant energy. The decreased AMP/ATP ratio reduces AMPK activity, activating mTORC1, which inhibits autophagy and downregulates LC3. This mechanism aligns with previous reports (50, 51). Treatment with tehranolide, however, elevated LC3-II/I levels. Similar effects have been observed with artemisinin, which induces autophagy by upregulating autophagy-related proteins such as LC3 (14, 42).

It should be noted that changes in LC3 expression, including LC3-II accumulation, are indirect markers of autophagy and do not alone provide definitive evidence of autophagy induction, as increased LC3-II may reflect either enhanced autophagosome formation or impaired degradation. Without direct autophagic flux assays, the current findings suggest autophagy involvement rather than conclusive activation. The study further investigated the role of SIRT1, a central regulator of autophagy (52), in tehranolide-mediated effects. Using sirtinol, a selective SIRT1 inhibitor, real-time PCR and Western blot experiments demonstrated that inhibition of SIRT1 reduced tehranolide-associated changes in LC3 and beclin-1 expression, supporting a potential involvement of SIRT1 in autophagy-related responses. Similar autophagy suppression by sirtinol has been reported in MCF7 cells (53).

Furthermore, research on artesunate, an artemisinin derivative, highlights autophagy regulation via the AMPK/SIRT1 pathway (54). Supporting this, BafA1, an autophagy inhibitor, prevented tehranolide-induced reductions in intracellular TGs, indicating that autophagy-related processes contribute to its lipid-modulating effects (55).

Findings from previous studies indicate that high glucose exposure (glucotoxicity) is known to induce apoptosis and hepatocyte damage (56), whereas autophagy is fundamental to suppressing apoptosis and maintaining liver tissue integrity (57). For example, globular adiponectin induces autophagy via AMPK/forkhead box O3A (FOXO3A) signaling to prevent ethanol-induced apoptosis (58), and ursodeoxycholic acid promotes autophagy and inhibits apoptosis by modulating Bcl-2/beclin-1 and Bcl-2/Bax interactions through AMPK activation (59).

In this study, to examine the protective effects of tehranolide against glucose-induced cytotoxicity, LDH release assays were performed with BafA1, rapamycin, and sirtinol. Rapamycin, a potent mTORC1 inhibitor, blocks this central negative regulator of autophagy, which normally suppresses the process under nutrient-rich conditions. By inhibiting mTORC1, rapamycin activates autophagy, promoting autophagosome formation and degradation of cellular contents (60). The LDH release assay demonstrated that exposure to toxic glucose concentrations (75 mM) induced significant membrane damage, whereas tehranolide markedly attenuated LDH release. Importantly, this cytoprotective effect was abolished by autophagy inhibition with BafA1, enhanced by rapamycin, and nullified by SIRT1 inhibition using sirtinol, underscoring the critical involvement of SIRT1-dependent autophagy in tehranolide-mediated cell survival. These findings highlight the necessity of finely tuned autophagic activity in determining cell fate under metabolic stress, consistent with the dual role of autophagy as either a protective mechanism or a driver of autophagic cell death when dysregulated (61).

In line with this autophagy-dependent cytoprotective profile, tehranolide also exerted profound effects on cellular energy and lipid metabolism. Suppression of key lipogenic genes, particularly FASN and SREBP-1c, coincided with a significant increase in p-AMPK, without altering total AMPK levels, indicating activation of the AMPK pathway. Activated AMPK is a central metabolic sensor that inhibits lipid synthesis, promotes fatty acid oxidation, and stimulates autophagy, while functionally interacting with SIRT1 to preserve cellular energy homeostasis and protection (62). Moreover, tehranolide elevated intracellular cAMP levels in an adenylate cyclase–dependent manner, an effect attenuated by the soluble adenylyl cyclase inhibitor KH7, which may indicate the activation of AMPK via cAMP as an upstream signal linking metabolic regulation to SIRT1-associated autophagic cytoprotection.

In addition to regulating lipid metabolism and autophagic cytoprotection, AMPK/SIRT1 signaling may also modulate inflammatory responses, which are closely linked to metabolic stress in hepatocytes. Inflammatory mediators such as IL-6, IL-1β, and TNF-α exacerbate hepatic steatosis (63), and autophagy disruption amplifies inflammatory responses (64). In this study, measurement of inflammatory cytokines showed that glucose-induced lipid accumulation in HepG2 cells led to an increase in inflammatory cytokines. The effect of glucose on cytokine upregulation in HepG2 cells has also been observed in previous studies (65). Tehranolide significantly reduced inflammatory cytokines in glucose-induced steatotic HepG2 cells. In vivo experiments showed that tehranolide is involved in modulating immune and inflammatory responses (16). Loss of SIRT1 expression in hepatocytes has been reported to trigger localized inflammatory responses, according to recent findings (10). Additional investigations are required to clarify whether activation of SIRT1-dependent autophagy accounts for the anti-inflammatory potential of tehranolide in hepatocellular models.

Collectively, the present study provides preliminary insights into the effects of tehranolide on glucose-induced lipid accumulation and autophagy-associated signaling in HepG2 cells. While pharmacological inhibitors were employed to support the involvement of the cAMP/AMPK/SIRT1 pathway, the lack of genetic approaches — such as small interfering RNA (siRNA)/short hairpin RNA (shRNA)-mediated gene silencing or overexpression of AMPK or SIRT1 — limits definitive causal interpretation. Accordingly, the proposed cAMP/AMPK/SIRT1-autophagy axis should be viewed as an associated signaling pathway rather than a strictly linear and causative mechanism.

Moreover, the glucose-induced steatotic HepG2 cell model used in this study does not fully recapitulate the multifactorial nature of MASLD, which encompasses additional contributors such as free fatty acid overload, lipotoxicity, inflammatory signaling, and oxidative stress. Although this model is suitable for dissecting molecular events related to glucose-driven lipid accumulation, its capacity to reflect the complex physiological and pathological conditions of the human liver remains limited. Finally, given that HepG2 cells are a hepatoma-derived cell line with altered metabolic characteristics compared to primary human hepatocytes, the translational relevance of these findings should be interpreted with caution. Therefore, the present results should not be considered definitive evidence of the therapeutic efficacy of tehranolide in liver diseases. Future studies employing genetic manipulation strategies, primary hepatocytes, and in vivo animal models of hepatic steatosis are warranted to validate these observations and to further clarify the molecular mechanisms and translational potential of tehranolide.

### 5.1. Conclusions

In summary, the current study provides preliminary evidence that tehranolide alleviates glucose-induced lipid accumulation in HepG2 cells. These effects are accompanied by increased expression of autophagy-related markers and modulation of the cAMP/AMPK/SIRT1 signaling axis, along with reduced production of inflammatory cytokines. While these findings suggest a potential involvement of autophagy-related pathways in tehranolide’s protective effects, further in vivo studies and genetic approaches are required to clarify the underlying molecular mechanisms and establish causal relationships.

## Data Availability

The data generated and analyzed during this study are not publicly accessible.
